# Identifying Prototypical Components in Behaviour Using Clustering Algorithms

**DOI:** 10.1371/journal.pone.0009361

**Published:** 2010-02-22

**Authors:** Elke Braun, Bart Geurten, Martin Egelhaaf

**Affiliations:** Neurobiology and Center of Excellence Cognitive Interaction Technology, Bielefeld University, Bielefeld, Germany; Freie Universitaet Berlin, Germany

## Abstract

Quantitative analysis of animal behaviour is a requirement to understand the task solving strategies of animals and the underlying control mechanisms. The identification of repeatedly occurring behavioural components is thereby a key element of a structured quantitative description. However, the complexity of most behaviours makes the identification of such behavioural components a challenging problem. We propose an automatic and objective approach for determining and evaluating prototypical behavioural components. Behavioural prototypes are identified using clustering algorithms and finally evaluated with respect to their ability to represent the whole behavioural data set. The prototypes allow for a meaningful segmentation of behavioural sequences. We applied our clustering approach to identify prototypical movements of the head of blowflies during cruising flight. The results confirm the previously established saccadic gaze strategy by the set of prototypes being divided into either predominantly translational or rotational movements, respectively. The prototypes reveal additional details about the saccadic and intersaccadic flight sections that could not be unravelled so far. Successful application of the proposed approach to behavioural data shows its ability to automatically identify prototypical behavioural components within a large and noisy database and to evaluate these with respect to their quality and stability. Hence, this approach might be applied to a broad range of behavioural and neural data obtained from different animals and in different contexts.

## Introduction

Animals behave in their environment accomplishing various tasks, like searching for food or partners. The analysis and comparison of animal behaviour, therefore, is necessary to understand their task solving strategies (e.g. in locomotion and flight control [1], [2]) and to identify the underlying mechanisms (e.g. genetic controlling [3], [4]). Before attempting to interpret behaviour, we have to describe relevant parts of it in an objective and quantitative manner. The identification of repeatedly occurring behavioural components is a generally applied approach to structure behavioural sequences of animals as well as of humans that often appear to be continuous (e.g. [5]–[9]). However, due to the complexity and variability of behaviour, it is a challenging task to identify those components in an objective way. In this paper we present one way how this can be achieved.

Behavioural scientists often define categories of behavioural components just by visual inspection dependent on the question to be answered [10], [11]. This kind of categorisation has to be done with care, because it significantly influences what data is collected, the collection procedures and, eventually, the success or failure of the analysis. In any case, much experimental experience is required to define the categories in a way that allows the currently observed behaviour to be classified unambiguously according to them. For example, Fentress and Stilwell [5] identified seven categories in mice grooming behaviour by visual inspection. These categories are, amongst others, *flurry of forelimbs below face*, *large synchronous but asymmetric strokes of forelimbs over top of head*, as well as *momentary interruption of active movement*, *with forelimbs at chest height*. However, the class description employed here leaves it up to the observer to decide, for instance, which strokes of the forelimbs are *large*. In order to get reproducible results it is desirable to quantify the class definitions by determining appropriate measurable characteristics, like, in the example here, the positions and the velocities of the forelimbs for quantitatively describing their movements. Calculating the corresponding values based on the behavioural data and taking the distributions of values within their value ranges into account leads to distinct accumulation points corresponding to prototypical behavioural components. Instead of assigning the a priori defined categories to the values, we propose to exploit the accumulation points within the value distribution of suitable characteristics for automatically determining precisely defined behavioural categories.

Selecting candidates for those characteristics depends on the aspired kind of behavioural description that is determined by the goal of the experiment. A description of behaviour is called ‘functional’ if it comprises the function or the consequence of the behaviour, like *grooming top of the mouse head*. In contrast, an ‘empirical’ description contains the structure, the appearance, temporal pattern etc. of a behaviour, as for example the description of the forelimb movements. Empirical quantities characterising movements may comprise the coordinates of an animal's position, its orientations, or its translational and rotational velocities, accelerations, etc. Generally, one has to select quantities that, on the one hand, provide valuable information about the behaviours and, on the other hand, are reliable, i.e. they can be extracted reproducibly, consistently and precisely from the experimental data. For instance, to automatically classify previously known phenotypes of *C. elegans* on the basis of their behaviour J.H. Baek et al. [12] successfully used several measurements ranging from form and size to velocity parameters. K. Hoshi et al. [13] uses the head and tail positions of *C.elegans* in image sequences in order to distinguish four typical locomotion states. The locomotion behaviour of *Drosophila melanogaster* is quantitatively described using trajectory characteristics like lengths, velocities, and turning frequencies for comparing different genetically modified strains (e.g. [14], [15]) or analysing the influence of drug treatments [16].

A special problem of selecting characteristic measurements for categorising behavioural data lies in the temporal aspect because behaviour often appears to occur as a continuous sequence. To identify behavioural components this sequence has to be segmented in a meaningful way. One commonly applied approach to this problem is to separate the segmentation from the categorisation step. This is done by using data sequences of definite and equal length [14]–[17] or by exploiting additional knowledge. For example, in [18], [19] the borders of meaningful movement segments are identified as points of time of zero velocities. Separating the segmentation step may simplify the following categorization, because a characteristic time course of a value during the whole segment can be exploited for classifying the segment. However, additional application dependent knowledge is necessary to perform the segmentation.

Without prior knowledge about the borders of meaningful segments the sequence is firstly divided into snippets corresponding to time steps of short and equal length. Categorization then takes place for each time step individually resulting in a sequence of category labels. If the time steps are short enough this sequence will provide subsequences of constant labels. They constitute segments of variable length that contain meaningful components of behaviour, as shown, for example, by G. Stephens et al. [1] while detecting basic shape types of *C. elegans* or by A. Galata et al. [20] for segmenting data from humans performing different exercise routines based on prototypical human silhouettes. Our approach also follows this idea of combined categorisation and segmentation, because it is generally applicable to different kinds of behavioural data.

Exploiting characteristic quantities, called *features*, for automatically determining categories is known in computer science as *unsupervised learning* [21]–[23]. The feature values calculated from the available database are assumed to provide distinct accumulation points, where each accumulation point corresponds to a category. *Clustering methods* ideally identify those distinct groups or *clusters* of feature values with strong internal similarities. Whether clustering is successful depends on the characteristic of the feature value distributions and on an appropriate parameterisation of the clustering procedure. Among the available algorithms the *k-means* approach is widely used due to its robustness and simplicity [23]. This approach was already successfully applied to behavioural data, for example, for classifying four behavioural phenotypes of *C. elegans* [24] or for determining action primitives used for steering the animation of an artificial game character [25].

After automatically determining clusters of feature values, we have to evaluate whether the clusters represent distinct and meaningful behavioural components. This implies the evaluation of the resulting cluster representatives with respect to their stability and quality in describing behavioural prototypes.

We applied and tested our approach to flight behaviour of blowflies, *Calliphora vicina*. By mounting coils on the heads of free-flying *Calliphora* and exploiting their magnetic induction, C. Schilstra and H. van Hateren were able to record large amounts of trajectory data containing the translational and rotational position of the fly's head within the 3D space during cruising flight [26]. Based on this data set the flight behaviour was divided into essentially two basic classes, saccades and intersaccadic intervals [27]. The proposed clustering procedure corroborates this finding, but, additionally, quantifies the results and answers the question, whether there are additional prototypical movements within the data set. The segmentation of behavioural sequences into prototypical movements constitutes the basis for further analysis of the individual movements and their correlation to external causal factors like visual stimuli or to internal causal factors like the neural activity in the brain controlling this behaviour. The latter aspects are beyond the scope of this article.

## Methods

The proposed approach for categorising behavioural data mainly consists of three steps: Feature selection, clustering and evaluation.

### Feature Selection, Extraction, and Normalisation

Before attempting to automatically categorise data, criteria need to be defined for discriminating different prototypical behaviours. These criteria, we call them *features* in the following, have to be objectively computable from the behavioural data. For each point in time of data acquisition we determine one value for each predefined feature from the behavioural data resulting in the extraction of a high dimensional *feature vector* for each point in time. This approach implies to leave out any information about the temporal sequence of the data points. We do this, because we want to use the categorisation of the individual data points to finally determine a meaningful segmentation in time, as already introduced above.

The selection of the characteristic *feature set* is the first and one of the most critical steps of the whole analysis. Ideally, we define the feature set in a way that makes the resulting feature vectors for different behaviours well separable from each other in order to allow the clustering algorithm to automatically detect this separation. Feature selection generally is determined by the experimental question to be answered and by the available data. For example, if spatial behaviour is to be analysed spatial coordinates and orientations of an animal may constitute valuable features, whereas with the goal to describe the dynamics of behaviour features such as velocities and accelerations may be appropriate.

In order to compare feature values we have to define a measure of their similarity or dissimilarity which strongly depends on the kind of involved features and their values. If feature extraction delivers categorical values, like red, green, blue, or binary ones, the definition of a measure of similarity is especially difficult (see e.g. [28]–[30]). Dependent on the application it might be possible to define numerical distances between the distinct values of a categorical variable. However, this discussion is beyond the scope of this article, since we here constrain ourselves on continuous numeric feature values, like we get for spatial coordinates, velocities or accelerations of a moving animal. For evaluating and comparing the (dis)similarity of each pair of high dimensional feature values we calculate the squared Euclidian distance within the n-dimensional feature space. This distance measure is in common use and computationally advantageous especially when applied to k-means clustering.

Generally, we need several features to discriminate prototypical behaviours. However, just taking into account more and more features without selection implies a high computational load for feature extraction and the subsequent clustering. Even more, noisy values may be obtained when calculating features that cannot be discriminated within the given database or cannot be extracted in good quality from the data due to experimental restrictions. Such noisy feature values may interfere with the separation within the feature space and may cause the clustering algorithm to fail. In order to evaluate and select a subset of relevant features without applying prior knowledge about the inner structure of the feature values *principal component analysis* (PCA) is in common use (e.g. [23]). PCA determines the axes of largest variability within the high dimensional feature values and thereby gives insight into linear dependencies between feature values which may give indications for possible dimensionality reduction.

After selecting the final set of features and extracting the corresponding values we have to take into account that the distance between two vectors of feature values is calculated as the sum of the differences between the individual feature values. These differences depend on the range of occurring feature values. To ensure that the differences corresponding to individual features contribute to the final distance value we have to normalise the ranges of the individual feature values. Apart from special application-dependent approaches, there are simple normalisation procedures that can be generally applied: Either the values of all features are normalised to a common value range, or the values of each feature are normalised individually to zero mean and standard deviation one [31]. The latter approach ensures comparability of the values without forcing the data with all outliers to one fixed range.

### Clustering

Clustering aims at identifying groups of similar data within the generally widely spread data. We apply the clustering method to our feature data. To identify groups of similar feature values clustering procedures use a suitable distance measure to the feature value vectors, as discussed above. The intention is to identify clusters that provide minimal *inner-cluster distances* and maximal *inter-cluster distances*. As a representative for each cluster a centroid is chosen by determining the feature vector that has minimal distance to all the members of the cluster. By renormalisation to the original feature values, the centroids become *feature prototypes*. Segmenting the temporal sequence of behavioural data by identifying subsequences of constantly assigned prototypes leads to segments containing *prototypical behaviour*.

In contrast to supervised classification, clustering approaches are applied, if the groups are not known in advance and, therefore, belong to the field of *unsupervised learning* in computer science. Without applying prior knowledge about classes the clustering process aims at discovering the inner structure of the data, resulting in objective and stable classifications. ‘Objective’ means that the same data processed with the same method leads to the same results. ‘Stable’ means that the results are invariant against variations of the special data, i.e. if, for instance, another set of data is used that was obtained by the same type of experiment [28].

Clustering techniques are widely used in many disciplines of science and accordingly many approaches have been developed (e.g. [28], [29], [31]). Two principally different clustering approaches can be distinguished: hierarchical algorithms and partitioning algorithms [30]. Hierarchical clustering either follows a splitting or an agglomerative procedure. The first starts with one cluster that contains all data and iteratively splits this cluster according to given criteria. An agglomerative hierarchical clustering starts with the finest granulation, i.e. each feature value vector builds its own cluster, and iteratively merges pairs of them by minimising the costs of merging via the distance to be bridged. The approaches of the second main group, the partitioning algorithms, start from a given group configuration describing a partitioning of the feature space and proceed by exchanging data elements between the groups. The partitioning of the feature space proceeds until a given end criterion is reached. Thereby, the assignment of a single data element to a group generally changes during the process, while in hierarchical clustering an assignment decision is fixed at the risk of false decisions based on outlier values and noise.

For our application of clustering a large amount of noisy behaviour-based feature data, we decided to use the most prominent representative of the class of partitioning algorithms, the k-means approach using Lloyd's algorithm [23]. This approach requires selecting the number of clusters in advance. For evaluating the selection of an appropriate number of clusters based on k-means clustering results several approaches exist in the literature that will be discussed and extended below. However, for applying k-means we need at least an idea about the range of cluster numbers to be tested to reduce the computational effort. To get this idea without prior knowledge about the number of meaningful clusters, we applied first an agglomerative hierarchical approach. For applications that provide this prior knowledge the hierarchical clustering step can be skipped.

Due to the basic idea of agglomerative hierarchical clustering, all possible numbers of clusters are built and the costs for merging and thereby reaching a special number of clusters are calculated. By analysing the slope of the cost function with decreasing numbers of clusters possible promising numbers of clusters can be identified by determining significant increases in merging costs. The evaluation of an increase to be significant thereby depends on the stage of the algorithm and has to be done in comparison to the neighbouring absolute costs. We should have in mind that this procedure is not suited for reliably clustering large and noisy data sets due to the extensive distance calculations and the local decision mechanism. Therefore, agglomerative hierarchical clustering will generally not be able to determine the appropriate number of clusters. Nonetheless, its application to parts of the data is appropriate to initially restrict the range of cluster numbers to be evaluated in detail based on k-means clustering results.

In the following two sections we will introduce the agglomerative hierarchical clustering and the k-means clustering algorithm. Readers who are already familiar with these approaches are encouraged to skip these sections.

### Agglomerative Hierarchical Clustering Using Ward's Criterion

Agglomerative hierarchical clustering starts with each feature value vector representing an individual cluster. Then the algorithm searches the two clusters that provide the smallest joining costs, merges them to form one new cluster and does so until all feature value vectors are agglomerated into one cluster. For determining the costs of joining two clusters we use *Ward's criterion*, which is one of several widely used criteria to be applied with agglomerative hierarchical clustering. It is based on the variance of the data within one cluster 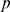, which is calculated as the sum of the squared Euclidian distances between the elementary feature vectors 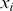 and the centroid 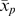: 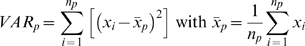
The variance of a new cluster generated by joining two clusters 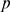 and 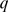 is:
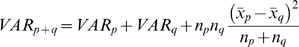
Ward's criterion for joining two clusters within the cluster hierarchy is to search for the two clusters that minimize the increase of the variance, which is given by the third summand.

When analysing the sequence of costs arising with more and more joining clusters according to Ward's criterion the costs are usually small in the beginning, where feature vectors are grouped that are very similar to each other. At the point where distinct groups within the data are forced to be joined, costs should increase significantly in comparison to neighboured values and thereby give a hint at a suitable number of clusters to be built from the given data.

**Figure 1:**
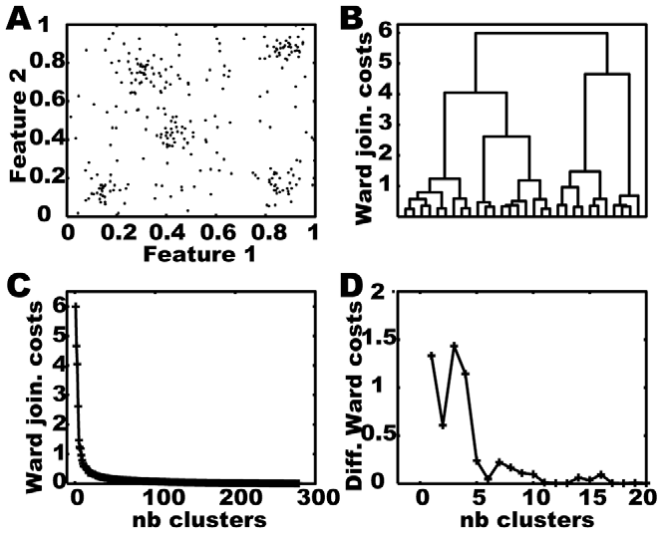
Hierarchical clustering approach. A) Artificial data of a two dimensional feature set. B) Dendrogram of applying agglomerative hierarchical clustering using Ward's criterion on the data shown in A. The x-axis indicates individual data points from A. C) Joining costs plotted against the number of clusters. D) Differential joining costs for the interesting range of number of clusters. Costs increase significantly if the algorithm groups the data in less than five clusters.

Figure 1 illustrates the agglomerative hierarchical clustering approach using Ward's criterion for a set of two dimensional vectors of artificial feature values (Figure 1A). Figure 1B shows the hierarchy resulting from clustering the data in the form of a so called *dendrogram*. The joining costs stay small with a decreasing number of clusters until four or less clusters are generated (Figure 1C,D). For this data the slope gives the clear hint that five clusters should be built.

The agglomerative hierarchical approach is suited only for small data sets due to its extensive distance calculations. Additionally, the approach locally clusters data points and shifts centroids according to the new cluster member. This very local approach is sensitive to noise because a small deviation within the data may change the sequence of clustering and thereby the intermediate centroids which themselves determine the next clustering step. Nonetheless, we propose to apply the hierarchical clustering approach to noisy behavioural feature data in order to constrain the promising range of cluster numbers, if there is no prior knowledge for this restriction available.

### K-Means Clustering

**Figure 2:**
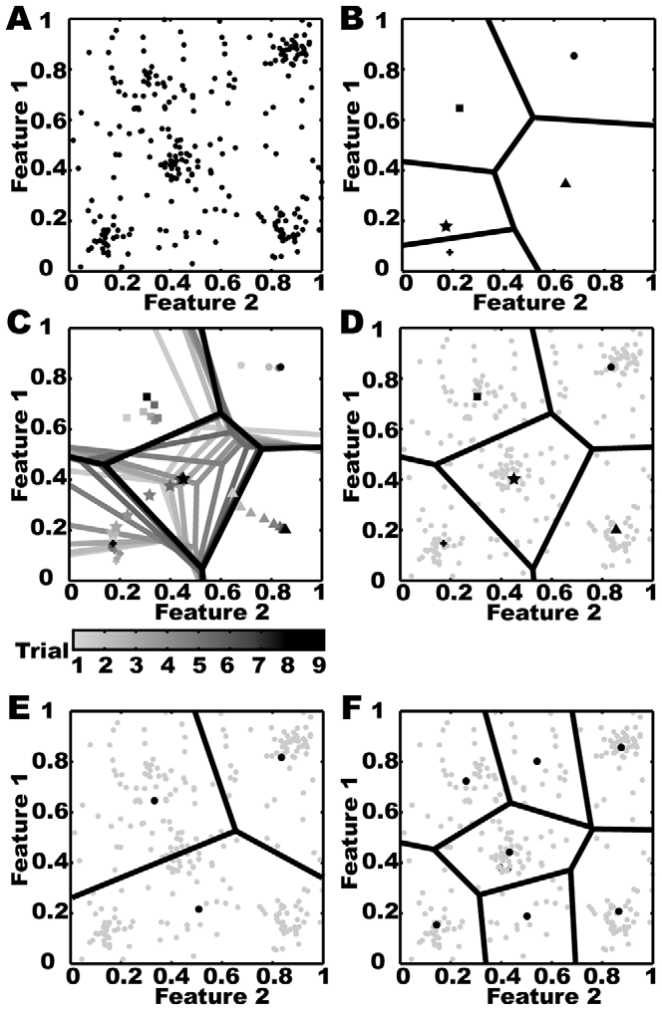
K-means clustering approach. A) Two dimensional artificial feature vectors to be clustered. B) Solid lines divide the feature space into Voronoi cells for the random centroid starting positions. Each of the cell's centroids is denoted by an individual marker. C) Voronoi plots of the nine steps needed by a k-means algorithm to find five stable clusters. The greyness of lines and markers indicates to which step of clustering they belong. D) The final clustering is shown in black above the data in grey. E,F) Results of clustering assuming an improper numbers of clusters.

K-means is the most popular partitioning clustering technique to be applied to large data sets [23]. It partitions the feature space into so-called *Voronoi cells* by determining k feature vectors to be cluster centroids and associating each part of the feature space to its nearest centroid according to a previously defined distance measure (Figure 2D). The choice of cluster centroids and thus the partitioning of the feature space is done in order to minimize the overall sum of distances between the feature values and their corresponding centroids. Using especially the squared Euclidian distance criterion, as for the agglomerative clustering described above, the generally formulated overall sum of distances to be minimised is given by the variance of the feature data, and each centroid becomes the mean value or centre of its assigned feature data:
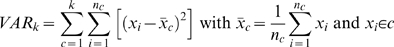
However, finding the global optimum is a np-hard problem and requires approximations like the iterative *Lloyd's algorithm* [23]. It iteratively improves the set of starting centroid candidates in order to find a centroid distribution that leads at least to a local minimum of the variance function. The algorithm is based on the observation that, as a consequence of using the squared Euclidian or variance distance criterion, the optimal centroids fall together with the mean value of the assigned data. The k-means clustering using Lloyd's algorithm applied to artificial two-dimensional feature values is shown in Figure 2.

Starting with any set of 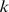 centroids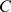, Lloyd's algorithm determines 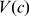 to be the Voronoi cell of centroid 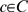, which is the set of feature vectors for which 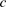 is the nearest centroid (Figure 2B). The centres of the data within the Voronoi cells are calculated iteratively as new candidates for the centroids, and new Voronoi cells are determined based on the current candidates (Figure 2C). These steps are repeated until predefined conditions for the convergence of the centroids and/or number of iteration steps is reached (Figure 2D). Lloyd's algorithm does not define the selection of the starting set of centroids. If there is no prior knowledge about the centroids, they are mostly determined by randomly selecting k feature value vectors from the data set (see [23], [28], [31] for detailed discussion of selecting strategies).

Although Lloyd's iterative approach makes the k-means problem treatable, the computational effort to calculate the distances necessary at each step of iteration is very high. Therefore, some accelerating approaches were developed in previous studies, which mainly increase the reusability of distance calculations instead of starting from zero at each iteration step. We applied the accelerating approach of T. Kanungo et al. [32] to our high dimensional behavioural feature data. It is based on the idea to firstly structure the data set within a tree. Each node contains one or more data points and additionally provides the number of the contained points and their mean value. For each node that contains a set of data points dependent nodes exist that contain parts of these data points together with the appropriate additional information. This tree structure accelerates the procedure to select for each data point at each step of the iteration the nearest centroid of the current candidate set. By exploiting the knowledge about the mean vector stored in each node centroids from the current set can be excluded to be considered for the current and all dependent nodes. The accelerated algorithm significantly simplifies the analysis of large amounts of high dimensional data but does not change the result.

Independent of the applied algorithm to calculate Lloyd's iteration, the resulting set of centroids may depend on the starting configuration, if there are local minima of the variance function. To ensure that the iteration reaches a significant minimum needed for further drawing general conclusions from the appropriate cluster centroids, repeated runs starting from different configurations are essential. We run Lloyd's algorithm with 1000 steps per run, which leads, together with a suitable threshold for convergence, to about 10 to 15 times selecting new random start positions.

Even for perfectly structured data the resulting centroids may not be meaningful due to an inappropriate choice for the number of clusters (Figure 2E,F). This choice has to be made in advance based on prior knowledge, or k-means has to be applied repeatedly using different values of k. For restricting the range of cluster numbers to be tested, we propose to apply agglomerative hierarchical clustering, as described above.

As a result of repeatedly applying k-means we get different clusters in dependence on k. Moreover, different centroids are obtained for each k when repeating clustering for different starting conditions, which is done to avoid meaningless local minima of the variance function. The different results have to be evaluated within an additional postprocessing step.

### Cluster Validation

The k-means clustering approach using Lloyd's algorithm delivers k centroids for a given data set. The centroids are placed within the feature space with the objective to minimize the sum of the variances within the partitions of the data that are associated with one centroid. Before drawing conclusions from the resulting centroid configuration we have to evaluate, whether the clusters defined by the centroids represent significant structures within the data set. So, we need objective criteria to evaluate the *quality* of clustering, i.e. how well the clusters match the data, and the *stability* of configurations resulting from different runs. The final centroid configuration has to be stable against repeated applications of the algorithm to a fixed data set with different initial conditions and should stay valid under variations of the data set.

#### Variation of the data set

Clustering results have to generalize from the specific data set in order to be reliable and replicable instead of representing just a special island solution. Therefore, we need to define suitable data set variations. Some recent approaches to evaluate clustering results are based on resampling the data by applying random selections of subsets of the data [33], [34]. In [35] P. Smyth favours a random selection of data points to form different subsets over partitioning the data into subsets of fixed size. He stated the main difference to be that for the random selection each data point is taken into account several times within different data constellations.

For our application of clustering sequentially recorded behavioural data, leaving out randomly selected data points, results in a kind of temporal subsampling of the data. Therefore, we decided to systematically leave out subsequences of data and cluster the remaining data. This procedure corresponds to analysing less data in the sense of fewer recordings of fewer individuals and smaller sequences of behaviour. Generally, the systematic leave out system may increase the sensitivity of results to periodicity within the data. This effect is minimized by leaving out very differently sized subsequences and by ensuring that each data point is involved in different constellations for clustering. We determine the size of the subsequences to leave out 10%, 20% or 50% of the data and cut them at 50 equidistantly distributed positions (each 2%) within the data set.

#### Stability

For a given fixed data set, centroid configurations directly correspond to data partitions. Different clustering results for different initial conditions can be compared with respect to stability by determining the similarity of the data clusters [28]. For each pair of data points it is tested, whether both points are assigned to the same or different partitions for two clustering results. In this way the similarity between resulting partitions can be estimated. The requirement to test cluster stability also for varying data sets leads to several approaches to extend the idea of comparing partitions resulting from non-equal, but overlapping data sets [33], [36], or even disjoint data sets [34], [37].

Instead of comparing data partitions, we propose to evaluate the stability of clustering on the basis of distances between resulting centroids. This approach is much simpler in the presence of large amounts of data and, additionally, a criterion is obtained for interpreting each centroid to represent a stereotypic behaviour. For calculating the distance between two sets of centroids with the same cardinality, we assign each centroid of the first set to one centroid of the second set. The sum of distances between the assigned centroids is taken as the distance between the two centroid configurations.

To assign the individual centroids of two sets to each other we demand each centroid to occur exactly within one assignment and determine the assignments to minimize the sum of occurring distances. For solving the matching problem we apply the so-called *Hungarian algorithm* (e.g. [38]) that efficiently delivers the optimal match between two data sets based on distance values given for each pair of data elements. We choose squared Euclidian distances for comparing the centroids because we also used this measure for generating the centroids during the clustering process (see above).

We evaluate the stability of two sets of centroids resulting from repeated runs of the clustering procedure based on the distance calculated for the matched centroids. To improve the comparability of the result we, finally, normalize this value for all centroids within each set and for the number of features each centroid contains. This normalised centroid-based stability measure can be used to validate the results of clustering runs with random starting positions, with a variable number of predefined clusters, with a varying data base and even with variable features. For evaluating more than two runs, the distances between all possible pairs of sets are calculated to determine the *mean set of centroids*, which is the one providing the smallest mean distance to the other sets. The mean distance between this mean set and the others is taken as a measure of instability. The mean error of the mean value is calculated based on the standard deviation divided by the number of trials.

**Figure 3:**
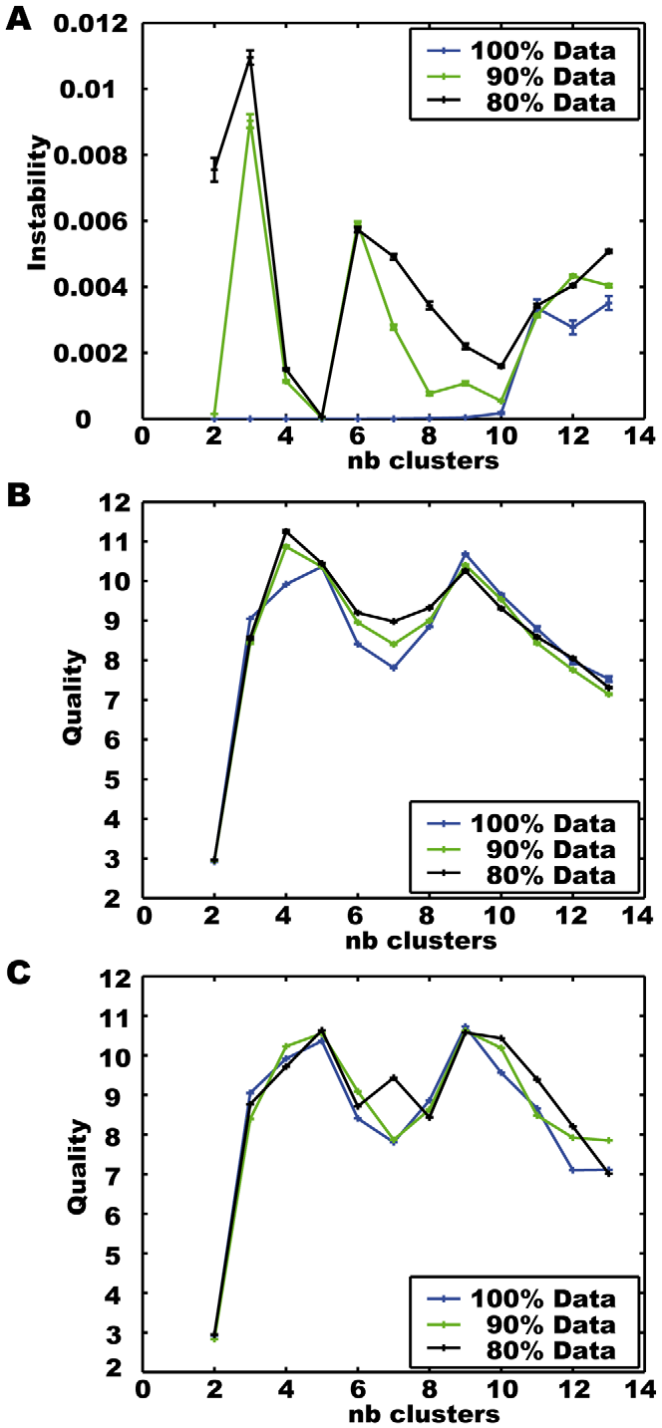
Evaluations for k-means clustering the artificial data of Figures 1 and 2. The number of clusters and the data sets are varied. A) Instability values. B) Mean quality values. C) Mean quality of mean set for each number of clusters, respectively.

Figure 3A shows the instability values for clustering the artificial data of Figures 1 and 2 in dependence on the number of clusters. In addition to the complete data set subsets containing only 90% and 80% of the data were clustered. 50 subsets for each condition were built by leaving out a subsequence of an appropriate size and shifting the leave-out position evenly over the whole data set. The centroids resulting from clustering the 50 reduced data sets for each condition are compared and their stability is shown. For the complete data set ten runs with different random start positions were analysed accordingly. Taking all data into account, the resulting cluster centroids for different starting configurations are stable for two to ten clusters. For the reduced data sets the instability increases on the whole, but has a minimum for generating five clusters. The minimum is even more pronounced when the data base is further reduced.

The stability of centroids under varying parameters of the clustering process is essential for their interpretation. However, stability alone is not sufficient for deciding about a meaningful number of clusters and whether the centroid configuration represents significant structures of the data.

#### Quality

A quality criterion should quantify how well the defined clusters represent distinct clouds of data points within the feature space. Many criteria to validate clustering results with respect to the number of clusters were suggested and compared [39], [40]. It should be distinguished between criteria that have to be applied during the clustering procedure to decide whether intermediate clusters should be merged or split and those that evaluate the final results of the clustering procedure. To validate our k-means results we are just interested in the latter ones. They work either with statistics about the membership of data points to clusters based on external knowledge or internal criteria (literature as in data variation), which both require high computational efforts. Or they exploit more general characteristics that are suited to describe a qualitatively good clustering.

These approaches determine, whether a centroid constellation leads to dense clusters of data that are well separated from each other. Dense clusters provide small variances within one cluster, where 
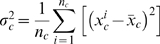
 is the variance of the data of cluster 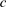, which is the mean squared distance between the data points 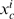 and their centroid 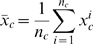. Well separated clusters are characterised by large distances between them. This outer distance is often calculated based on the centroids of each pair of clusters 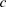 and 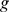 as 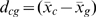. Qualitatively good clusters should provide small inner and large outer distances, which can be combined to one criterion by relating the two values. Kanungo et.al. [32] calculate one index for the whole clustering result as: 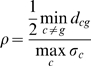, while Coggins and Jain [41] determine the separation of each individual cluster to be: 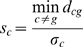. For our application, we use the clusterwise criterion with respect to our goal to interpret and evaluate the individual centroids and clusters as distinct components of behaviour. For simplicity we further use the squared index as our quality measure 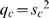, which changes the absolute quality values but does not make any difference in comparing values from different runs. We take the mean cluster quality 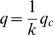 as an index for the quality of the whole clustering in order to compare results from differently parameterised runs.

Figure 3B shows the quality calculated for clustering the artificial data of Figures 1 and 2 in dependence on the number of clusters and for varying datasets. As for the stability measurement, in addition to the complete data set subsets containing 90% and 80% of the data were clustered. For the complete data set ten runs with different random starting configurations were analysed, while for the reduced data set conditions, the clustering results based on 50 different data sets for each condition are compared. The depicted quality value is the mean value for all sets resulting from one condition. For the complete data set the quality index reaches its global maximum for nine clusters, another local one for five clusters. However, for the reduced data sets we get the global maxima for four clusters, while the quality for five clusters is similar to that for nine clusters. This means that among the reduced data sets there are ones that provide four clusters of better quality rather than five clusters. These four cluster solutions for the reduced data set have even higher quality than the five clusters obtained with the complete data set. However, since four clusters do not lead to stable results for the reduced data sets, these isolated results of good quality for special data sets do not represent general significant structures of the data. For calculating the stability we determined the mean set of centroids for each number of clusters and size of the data base (see before). Assuming that these mean sets of centroids are best suited for representing the data, we calculated their quality, which clearly provides a maximum with five clusters, as shown in Figure 3C. Combining the quality and stability criterion leads to five meaningful clusters for this data.

**Figure 4:**
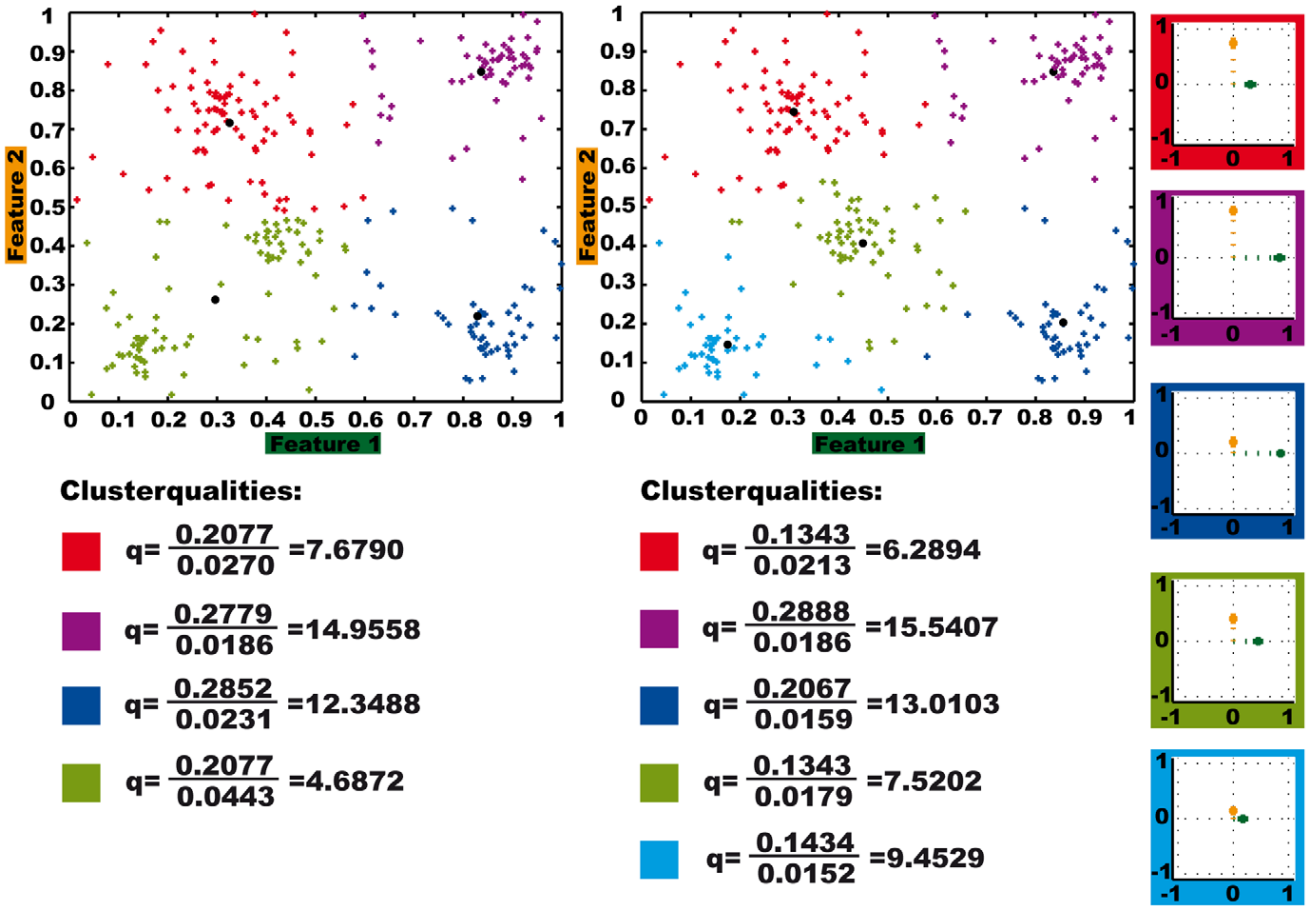
Quality values and centroid visualisation for individual clusters within the complete artificial data set. Clusters result from determining the mean set of four and five centroids, respectively. Visualisation of the five centroids as modified star plots, see text for explanation.

Besides validating the clustering result as a whole, we designed the quality criterion to evaluate individual clusters. Figure 4 shows the quality values for the mean set of centroids for determining four and five clusters, respectively, based on the complete artificial data. Generating five clusters instead of four decreases the variances within the clusters (Figure 4, lower part). In all of the clusters, except the red one, these decreases overcompensate the also occurring decreases in the outer distances between the clusters, resulting in higher cluster qualities. The quality values generally differ for individual clusters. For our application of clustering behavioural feature data and using the centroids for identifying prototypical behaviours this quality measure allows us a differentiated evaluation of the individual feature prototypes and the resulting prototypical behaviours.

In summary, the quality and the stability criterion in combination constitute a reliable tool for validating clustering results. Thereby the stability of results constitutes the prerequisite for further interpretation because it insures reproducibility and indicates that the results generalise from the concrete dataset. Stable results can be compared to each other by evaluating their quality.

### Visualization of Centroids

The centroids represent, where required after renormalization, feature prototypes. For a closer look at the prototypes a visualisation of the high-dimensional centroids is necessary, that depicts the values within the feature value space and allows us to easily compare the individual prototypes. Several visualisation methods for high-dimensional data are in use (e.g. [28], [31]) that are based on generating suitable two- or three-dimensional views on the high dimensional data cloud or showing the high-dimensional content of individual data points. For visualizing the centroids we use a representative of the latter class, the *star plot* [31], in a somewhat modified way.

A star plot of a centroid is a two dimensional star-shaped diagram with one ray for each feature, where the length of each ray is proportional to the corresponding feature value and the rays are drawn equi-angular around the centre. Positive feature values are depicted in the upper half of the star, while negative feature values occur on the corresponding ray with the respective opposite orientation. We modify the star plot by introducing colour for better discrimination of the individual rays and mark the end of each ray with an error bar indicating the standard deviation of the centroids mean value. For better visibility of this error coding we leave out the lines originally connecting the centre with the end of each ray (Figure 4, right column).

The artificial data set as well as the software used for the analysis and plotting the results that extends Matlab (R2009b) functionality is given in the supporting material S1.

## Results

We applied the clustering approaches to semi- free flight trajectories of the blowfly *Calliphora vicina* [26]. The clustering procedure delivers a set of feature prototypes that we use to determine a segmentation of the trajectory into repeatedly occurring prototypical elementary flight movements. Flight behaviour of *Calliphora vicina* was previously analysed in [27], [42], [43]. Our new method confirms these results and, in addition, differentiates the behavioural components characterised previously on the basis of visual inspection of the data.

### Database

We analysed head positions and gaze direction during cruising flights of *Calliphora vicina* that were recorded by C. Schilstra and J.H. van Hateren in a flight arena of about 40cm×40cm×40cm size surrounded by a Helmholtz coil. Small sensor coils were attached to the fly's head that induce voltages during motion within the surrounding magnetic field. The signals allow determining the three-dimensional position and the three-dimensional orientation of the fly's head with a temporal resolution of 1ms (for details see [26]).

The recorded flights range in their length from 1s to 24s. We selected for the clustering analysis all flights of at least 3s duration to ensure the data to contain cruising flight instead of starting and landing behaviour. This selection results in a data base containing 556343 data points, corresponding to about 556s of flight.

### Feature Extraction and Normalisation

**Figure 5:**
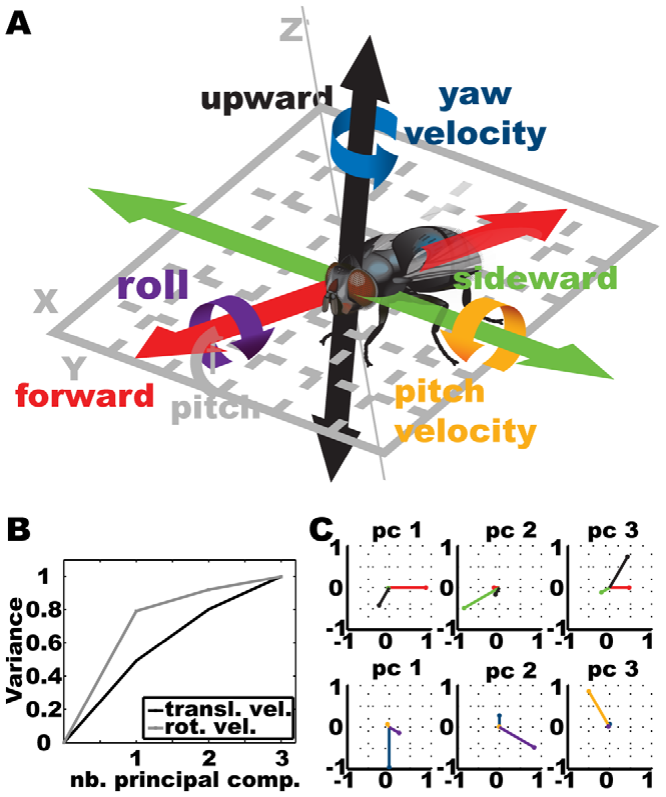
Calculating fly head velocity features. A) The fly head fixed coordinate system used for calculating three translational and three rotational velocities. B) PCA analysis of the two different sets of Calliphora head velocity data. Part of the covered data variance in dependence on the number of principal components taken into account. C) Visualisation of the principal components sorted in decreasing order of variance content, each.

As discriminative features for distinguishing different prototypical movements from the raw six dimensional trajectory data we selected the translational and rotational velocities measured within a fly-centred coordinate system. Generally, velocities are the most prominent features to describe movements of rigid bodies and the fly-centred coordinate system ensures the velocities to be independent from the location and orientation of the head within the arena. For calculating the velocities within the fly's coordinate system we transform the difference between the trajectory data at time 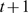 and 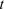 into the fly-centred coordinate system at time 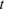 and determine the forward, sideward, and upward velocity as well as the yaw, pitch, and roll velocity as shown in Figure 5A.

The original trajectory data is noisy due to the experimental procedure. We, therefore, smoothed the original data by applying two times (forward and backward) a Butterworth filter of order two with a relative border frequency of 0.1. The filter parameters were chosen to be suitable to just marginally influence the trajectory slope and thereby the information content of the velocity data while strongly suppressing noise in the velocity data.

The translational and rotational velocities serve as features for the clustering process. As discussed above, the feature extraction step aims at generating feature values that are similar for similar movements and are separated for movements to be distinguished from each other. Ideally dense clouds of data are well separated from each other and spread over the feature space to be automatically detectable by the clustering process. For our application of categorizing trajectory data we should keep in mind that velocities change continuously between prototypical values. Those smooth transitions constitute noise for the clustering approach. However, the difficulty to handle transitions between more or less stable states exists independent from the selected features if we do not apply prior knowledge about the segmentation of the trajectory.

Extracting the velocity data from the filtered trajectory data delivers a six dimensional feature value vector for each trajectory point. Concerning the reduction of the feature set to the most variant, which are assumed to be the most relevant features, we applied principal component analysis (PCA) to the three dimensional translational velocities and the three dimensional rotational velocities independently. Figure 5B shows the variances within the two data sets as it is divided over the three principal components, respectively, and the corresponding principal components as they are composed of the original features visualized as star plots. For the rotational velocities the first principal component takes about 80% of the total variance, while the remaining two take about 10% each. The first component is dominated by the yaw velocity, the second by roll and the last by pitch. For the translational velocities the variances are spread wider over the three components with the first containing less than 50% of the total variance. The first component contains a mixture of forward and somewhat less downward velocity. This velocity combination, measured within the fly-centred coordinate system and the fly being pitched relative to the ground, corresponds roughly to horizontal flight. The second translatory principal component contains sideward velocity, while the last contains the counterpart to the first with dominating upward and less forward velocity.

The results of the PCA show that there are no feature combinations within the rotational and translational velocity groups that represent sufficiently well in lower dimensions the variances within the data. Therefore, we did not reduce the feature dimensions for our special application.

To ensure the comparability of the translational and rotational velocity values for clustering, we normalise the values for each kind of velocity independently to zero mean and standard deviation one.

### Hierarchical Clustering for Constraining the Number of Clusters

Applying agglomerative hierarchical clustering using Ward's joining criterion on the *Calliphora* head velocity feature data constrains the range of numbers of clusters to be built by the following k-means clustering. Since the hierarchical approach is, due to its extensive distance calculations, only suited for small data sets, we randomly selected three sub sequences from the large database each containing 5000 data points and clustered them individually.

**Figure 6:**
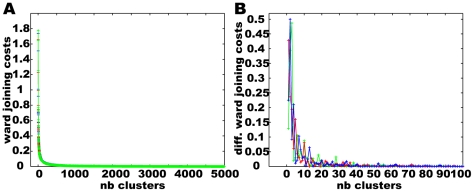
Hierarchical clustering of normalised Calliphora head velocity data. Three different data segments containing 5000 data points each were clustered using Ward's joining criterion. A) Joining costs in dependence on the remaining number of clusters. B) The deviation of the cost function for the most interesting range of fewer than 100 clusters.

Figure 6 shows that the joining costs for the three data sets differ only slightly. A distinct number of clusters cannot be identified in this data. On the one hand, the data fragments may to be too small to be representative. On the other hand, the approach is, owing to its focus on local data characteristics, sensitive to the noise contained within the experimental behavioural data. However, based on hierarchical clustering we selected cluster numbers between two and 50 for k-means clustering.

### Validating K-Means Clustering Results and Determining Suitable Numbers of Clusters

**Figure 7:**
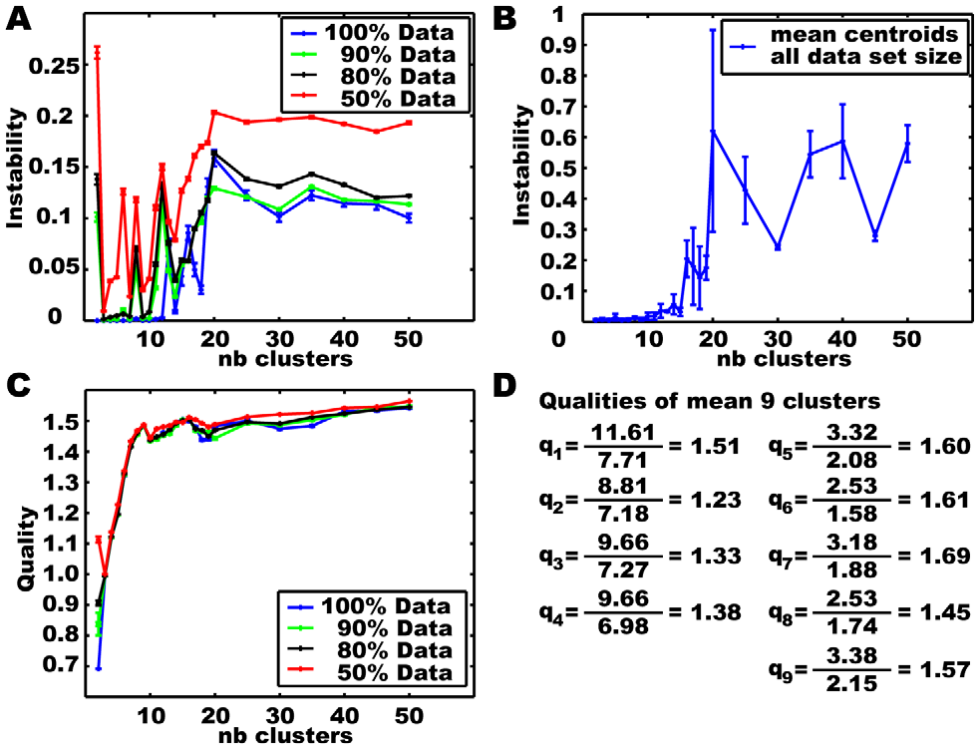
Criteria for validating the k-means clustering results for the normalised Calliphora head velocity data. Instability A) within and B) between data set configurations and C) quality of clustering results in dependence on the number of clusters for varying data sets. D) Individual quality values for mean nine centroids of the complete data set.

Given a range of suitable numbers of clusters we repeatedly applied k-means clustering to the whole velocity feature data as well as to the different sets of reduced data. For validating the clustering results we calculated the instability and quality for the resulting centroid configurations (Figure 7). The instability analysis (Figure 7A) reveals that, independent of the special data base, several numbers of clusters below twelve lead to stable results. Clustering the complete data set with different random starting positions leads to very small instabilities (<0.003) for the whole range from two to twelve clusters. In contrast, the instability curve calculated for centroid configurations based on varying reduced data sets provides clear local minima and maxima within this range of cluster numbers. The obviously instable points at six and eight clusters are caused by two distinct centroid configurations arising for variations within the data. Hence, it is necessary to validate the stability of the centroid configuration against varying data bases before generalizing the results and drawing conclusions from it. So far, we compared the centroids resulting for either the complete data or one of the reduced data set configurations. What is left to do is the analysis, whether stable centroids occur across the data set conditions. The instability for the different mean sets of centroids again is very small for up to ten clusters and only increases significantly if more clusters are generated (Figure 7B). From the instability analysis promising numbers of clusters are three, four, five, seven and nine. However, the quality criterion (Figure 7C) suggests the choice of nine clusters, because here the mean quality has a local maximum. Although the mean quality increases slightly with larger numbers of clusters, more clusters are not suitable for the data because of their instability.

The quality values corresponding to the individual clusters of the mean set of nine clusters for the complete data set are shown in Figure 7D. The mean quality for all clusters is 1.49. We get four clusters that provide large variances within their assigned data (inner distance), but also large (outer) distances to the nearest neighbouring centroid. The five other clusters provide significantly smaller variances but are also closer to their neighbours. The quality values for the two types of clusters are approximately the same.

### K-Means Clustering Results

**Figure 8:**
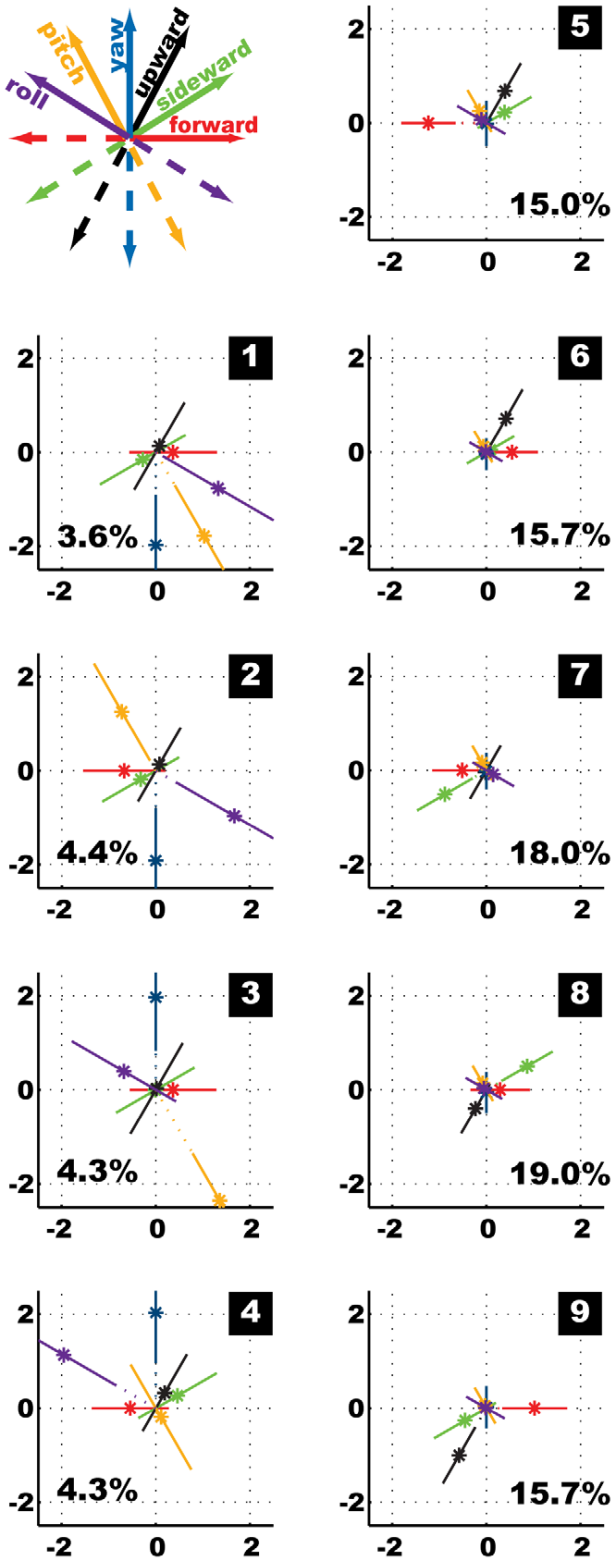
Mean set of nine centroids calculated based on the normalised complete data set for Calliphora free flight head velocity data. The part of the data in percent assigned to the individual centroid is given with each centroid.

**Figure 9:**
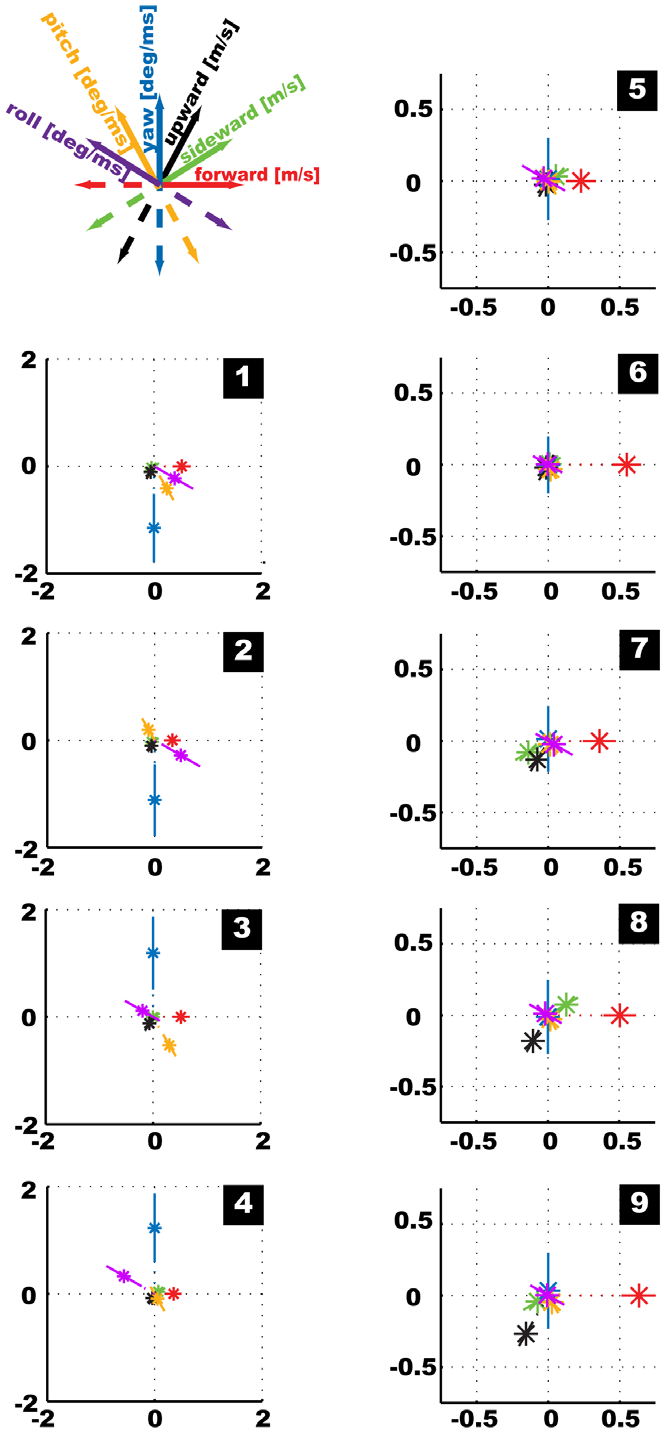
Mean set of nine velocity prototypes for Calliphora head data. Feature values are accomplished with physical units: m/s for translational, deg/ms for rotational velocities. Note the different scales for the rotationally and translationally dominated prototypes.

We identified nine clusters to represent the most suitable structure of our behavioural feature data (Figure 8). Amongst the resulting centroids we can distinguish one group comprising four centroids (Figure 8, 1–4) that are dominated by the normalised rotational velocities. A second group (Figure 8, 5–9) comprises the remaining five centroids with virtually no rotations but only translations. Each of the centroids dominated by rotational velocity features represents about 4% of the data, summing up to 16.62% for all of them. Within the other group the two centroids containing large normalised sideward velocities represent 18% and 19%, respectively, and the remaining ones each about 15% of the data. Hence, translational prototypes occur much more frequently than the rotational ones. Furthermore, the absolute values of the prototypical normalised rotational velocity features are higher than the translational ones, even though all original velocity data was normalised equally to zero mean and standard deviation one before clustering. The rotational velocity features mainly assume either rather large (positive or negative) values or are close to zero. In contrast, the translational velocity features within the centroids take additionally intermediate values. This finding indicates different characteristics for the distributions of the rotational and translational feature values. The four clusters dominated by rotational velocities provide large inner variances, but also large outer distances to their neighbours (Figure 7D). To analyse the centroids further we renormalize them to get the feature prototypes with their respective physical units, i.e. the translational and rotational velocities of *Calliphora* heads measured in m/s and deg/ms, respectively (Figure 9).

The separation of the nine prototypes into groups dominated by rotational and translational velocities, respectively, is in agreement with previous conclusions that flies tend to decouple translational and rotational movements by their saccadic flight and gaze strategy, thereby simplifying visual information processing by the nervous system [2], [42]. The prototypes dominated by rotational velocities provide yaw velocities with mean absolute values of about 1100 deg/s; roll and pitch velocities are smaller (100 to 660 deg/s) but also significantly different from zero. The mean rotational velocities of the other prototypes are close to zero. These velocity values allow us to identify four saccade and five intersaccade prototypes within our data.

Two of the saccadic prototypes correspond to left turns, the other two to right turns, respectively. Yaw and roll velocities are coupled: a left turn (positive yaw velocity, see Figure 5A), is accompanied by a right/clockwise roll (positive roll velocity, Figure 5A) and vice versa. The prototypes with similar roll and yaw velocities mainly differ in their pitch velocities; we find negative (head up) as well as positive (head down) pitch velocities. These head velocity combinations differ from the ones expected for the body of the fly. Hence, blowflies fly their turns as so-called banked turns (see also [27]), as also aircrafts do. This means that turns are flown as a combination of yaw, pitch and roll movements, instead of just applying yaw movements like cars on the street do. In our fly-centred coordinate system a banked turn of the body would corresponds to a combination of yaw with pitch up (negative velocity) and roll of opposite sign. For the head we find different combinations of yaw and roll and more variations for pitch velocity. This can be explained by the fly compensating for body roll and pitch in order to keep its head orientation to be as horizontal as possible for stabilizing gaze. The four prototypes representing saccades all contain also translational velocities, mainly forward velocities of about 0.4 m/s.

The five prototypes characterising intersaccades are dominated by the forward velocity, which takes values from 0.23 m/s up to 0.64 m/s. The forward velocities are coupled with downward velocities of 0.02 m/s up to 0.31 m/s. The combination of forward and downward velocities within the fly-centred coordinate system leads to horizontal movement of the head in the flight arena, if it takes a non-zero negative pitch angle. The prototype that combines the largest forward velocity (0.64 m/s) with the largest downward velocity (0.31 m/s) describes horizontal flight with a pitch angle of −26 deg. This pitch angle corresponds well to the mean pitch angle within the original trajectory data of −23 deg. Sideward velocities occur symmetrically within the prototypes. This is expected, because neither direction should be preferred over the other during sufficiently long sequences of cruising flight. Two prototypes contain the maximal sideward velocities of about 0.15 m/s, positive and negative, respectively, combined with intermediate values for forward and downward velocities.

### Segmentation of Behavioural Sequences into Prototypical Movements

**Figure 10:**
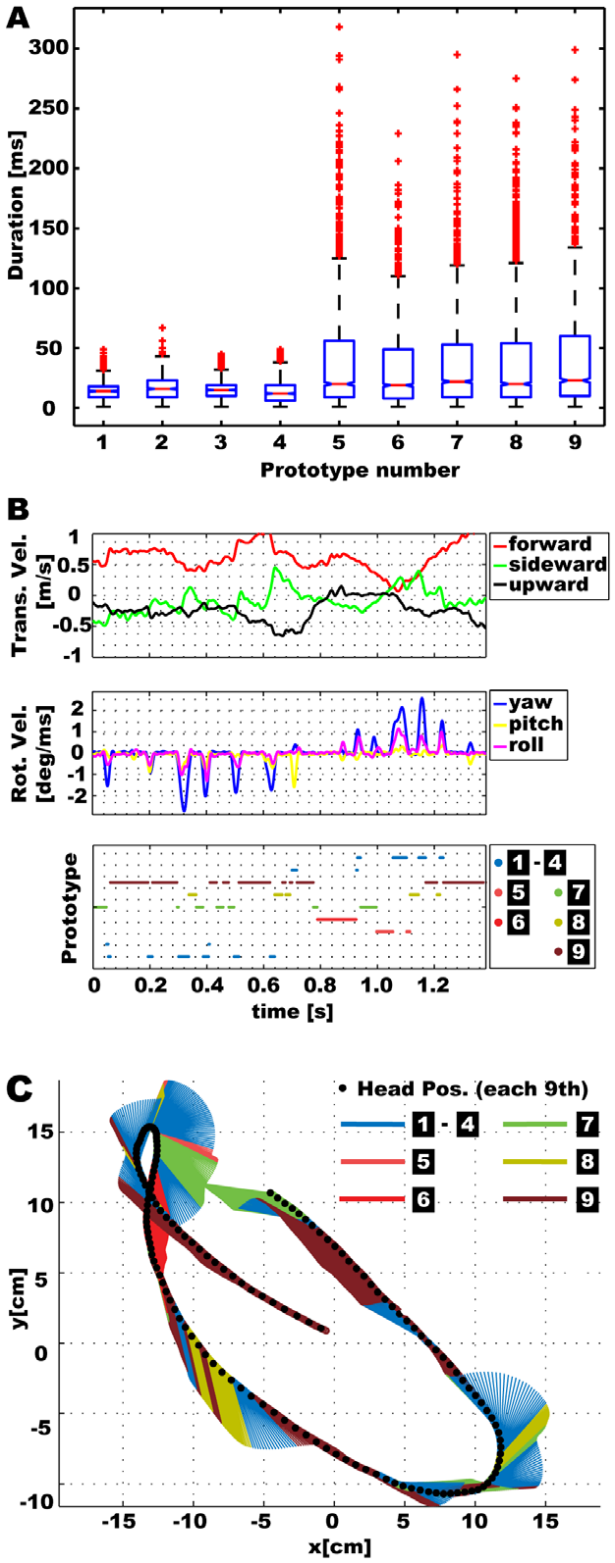
Segmentation of behavioural sequences into prototypical movements. A) Overall occurring lengths of prototypical movements for the individual prototypes. B) Velocity data and individually assigned velocity prototypes (prototype numbers as in Figure 9) for an exemplary part of a fly head trajectory. C) Segmentation of the example trajectory into prototypical movements. For better visualisation the trajectory is projected into two dimensions, just yaw rotation is shown, and the four saccadic prototypes are summarized resulting in six remaining differently coloured prototypes.

The velocity prototypes result from clustering the velocity data sets that are calculated from each two sequential trajectory points independent from each other. However, the trajectory delivers a sequence of velocities that can be assigned to a sequence of prototype indices. Detecting subsequences of constant indices allows the segmentation of the trajectory into prototypical movements, where each subsequence length corresponds to the duration of the prototypical movement. Figure 10A depicts all the occurring durations of the prototypical *Calliphora* head movements and shows that saccades are shorter, on average, and less variable in their duration than intersaccades. For saccades few sequences are longer than 20ms, while for intersaccades about half of the sequences take longer than 20ms. This finding confirms the saccadic flight style and gaze strategy that aims at minimizing the duration of rotational head movements, while the intersaccadic interval has a duration of some tens of milliseconds [2], [27].

Figure 10B and C shows the results for an exemplary part of a trajectory, which provides, for the longest time, prototypical movements of considerable duration. Occasionally all prototypes do also occur for very short durations (Figure 10A). This is the result of uncertainties in classification. We should keep in mind that reducing the complexity of behaviour to few prototypical components inevitably omits many details. Variations within individual velocities compared to the velocity prototypes lead to uncertain classifications, which results in faster transitions between prototypes (Figure 10 B,C, intersaccadic interval beginning at about 400ms) or even changes from point to point (data not shown). These uncertain classifications also occur for saccades, because all saccadic prototypes contain large yaw velocities accompanied with smaller pitch and roll velocities, while also saccades occur characterised by just one or combinations of two rotational velocities (Figure 10B). Additionally, transitions between two stable prototypes may provide velocities that fit best to a third prototype, as happened in the short sequence shown in yellow just before the last saccade of the example data at about 1210ms. In spite of these difficulties, prototypical movements of considerable duration indicate a meaningful classification and deliver an appropriate segmentation of the trajectory into prototypical behavioural components.

## Discussion

We determined prototypical movements from flight trajectories of the blowfly *Calliphora* as an application of a new approach for objectively classifying behavioural data. This approach automatically identifies behavioural components by applying appropriately parameterized k-means clustering to a quantitative representation of behavioural data in the form of high dimensional feature values.

The selection of suitable features is guided by the designated behavioural description, which depends on the question to be answered. Generally, features have to be selected to deliver feature values that can be extracted reliably from the experimental data and that are valid for distinguishing those behavioural components from each other that are relevant for answering the experimental question. Feature selection and the extraction of feature values determine the kind of categories that can be identified and thereby which aspect of the behavioural data is to be analysed. For example, for distinguishing prototypical movements of the *Calliphora* head we selected velocities as features and calculated their local values from the experimental trajectory data, while other aspects, like their spatial location, do not play a role for discrimination. The issue of selecting and extracting suitable feature values has to be addressed individually for each type of experiment.

In contrast, the approach for automatically identifying categories follows the general strategy to detect significant structures within the distribution of feature values. This strategy does not change with the individual set of selected features as long as the feature values are continuous numeric values that are normalized to zero mean and standard deviation one in order to allow for the quantitative comparison of the individual feature values as it is done by the general purpose k-means clustering approach implemented as Lloyd's iteration.

The iterative k-means locates the given number of k clusters within the noisy feature values. For determining a suitable number of clusters k the investigator has to test different numbers with k-means and evaluate whether the resulting clusters represent significant accumulation points of the data. This is a time consuming procedure if one has to test a large range of possible numbers of clusters. For abbreviating this, the range of suitable numbers of clusters can be restricted by applying prior knowledge about the behaviour to be analysed, if available. Generally, without such knowledge, we propose the application of a hierarchical clustering approach for identifying with less computational effort at least a range of suitable cluster numbers. For a restricted range of clusters numbers we calculate the k-means clusters and then apply criteria for evaluating whether the clusters represent significant accumulation points of the data. These structures are generally significant, if they lead to distinct clusters, if they stay stable for different random starting positions of the iteration, and if they generalise from the given data set to varying data sets.

We defined two criteria for the quality and stability of clusters, respectively. Analysing these criteria for varying data sets and cluster numbers allows us to identify sets of clusters that represent well the accumulation points of the feature values. The applications to artificial and real behavioural data show that we cannot expect both criteria to identify just one number of clusters to be most stable and of highest quality. Instead, quality generally increases with increasing number of clusters, because more clusters may better represent the details of the feature value distribution. However, usually instability increases accordingly, because dependent on the starting positions and the specific composition of the data base different details of the distribution become important leading to different cluster configurations.

To identify significant behavioural components that are independent from algorithmic details, like different random starting positions, and from the special properties of a given behavioural data set, we consider stability as a prerequisite for further analysing a set of clusters. Among the stable cluster configurations we select the one providing the best quality in representing the feature values within distinguished clusters. The evaluation determines, on the one hand, the number of clusters that leads to the best representation of significant structures in the data, and, on the other hand, indicates quantitatively the quality of this representation. Even for applications, where the number of classes seems to be clear, like the different *C.elegans* phenotypes used in [12], [24] or their locomotion states addressed in [13], the objectively determined classes that provide the best representation of the data can be useful to confirm the prior knowledge or improve it, if different categories occur within the data.

Feature values are calculated from the sequence of behavioural data by extracting one set of values per time step, i.e. one millisecond for the *Calliphora* data. Individually categorising these values per time step makes, on the one hand, the categorisation task more difficult but, on the other hand, delivers a categorisation without making prior assumptions about the temporal characteristic. If the independent categorisation of short time steps according to feature prototypes is successful in the sense of delivering meaningful behavioural classes the following identification of sequences of invariant prototypes deliver a segmentation of the behavioural sequence into prototypical components, which allows us investigating the temporal characteristics of the individual components. The evaluation of the durations of the components serves as a criterion for this success and clearly depends on the application, namely the kind of behaviour to be analysed. It can be assumed that investigating the temporal characteristics of behaviour instead of segments of previously defined length [14]–[17] might reveal additional insights in behavioural control. The clustering approach is more generally applicable in comparison to those relying on application dependent knowledge for segmentation, like zero velocity points as used in [18], [19].

The clustering of feature values together with the evaluation step delivers a set of cluster centroids that represents feature prototypes. This set of prototypes is suited for automatically categorising appropriate behavioural data, which clearly provides advantages in comparison to the common way of defining classes before starting the analysis. Even, if prior knowledge about the behaviour leads the selection of appropriate features the proposed clustering approach is able to automatically deliver a suitable quantitative description of behaviour.

By categorising behavioural data into few prototypes we do not take into account detailed variations of behaviour within the classes. This classification is too coarse for synthesising behavioural sequences, as it is done in [25] for artificial game characters using several hundreds of clusters. However, the few prototypes reduce the complexity to few general components that reveal the structure of behaviour. Given the classification into the prototypes allows us to investigate the variations within each prototype in order to analyse the influence of internal or external parameters.

The prototypes resulting from analysing *Calliphora* head trajectories show, in accordance with previous results [2], [42], that cruising *Calliphorae* show a saccadic flight and gaze strategy which means performing purely translational movements disrupted by short and fast manoeuvres dominated by rotations. Within the set of determined prototypes we can separate those that are characterised by high rotational velocities from those that describe virtually pure translations. Saccadic movements are shorter than intersaccadic ones. In addition to this distinction, our cluster analysis delivers nine velocity prototypes that constitute the basis for quantitatively describing prototypical movements as behavioural components.

The analysis of *Calliphora* head movements constitutes a successful application of the proposed approach for automatically classifying behavioural data. Clustering of suitable feature values and evaluating the results with respect to quality and stability turns out to be a robust method for determining behavioural components within large and noisy data bases. Due to the standardized method, prototypical components resulting from different behavioural contexts, including different environments, different tasks, and also different species can be compared to each other in order to investigate the influence of external factors on behaviour.

We applied in a parallel study the clustering approach to trajectory data originating from the hoverfly *Eristalis tenax* and were able to quantitatively describe their flight behaviour (Geurten et. al. submitted). Hoverflies reveal significantly more variance within the prototypical movements in comparison to *Calliphora*, including, among others, a prototype containing both translational and rotational velocities close to zero as is characteristic of hovering. Part of the prototypical movements of *Eristalis* was found to differ in two flight arenas of largely different size. In experiments with honeybees (*Apis mellifera*) navigation performance in environments with different visual landmark configurations around a feeder was tested. Our clustering approach revealed that the spatial distribution of particular velocity prototypes depends on the distance to visual landmarks (Braun et. al. in preparation).

Beyond insect locomotion our approach may well be applied to other areas of quantitative behavioural analysis, as, for example, for characterising the influence of genetic modifications [3], [4] or of drug treatment on behaviour [9], [16]. In this type of experiments the automatic identification of behavioural components may reveal new insights into behavioural differences that support the detection of the underlying control mechanisms. The segmentation of behavioural sequences into prototypical components additionally allows relating behaviour, for example, to visual input and to the corresponding neuronal activity by appropriately designed stimuli [44]. We just started to apply the proposed clustering approach to identify prototypical components within neuronal data helping to understand the structure of neuronal information.

Generally, prototypical components constitute the basis of structurally describing more complex sequences as rule-based sequences of these components [5], [45], [46]. The derivation of probabilistic rules based on the transition probabilities between individual prototypes is the stringent extension of the presented approach for describing complex sequences (Geurten et. al. submitted).

## Supporting Information

Supporting Material S1 Artificial data set and matlab function toolbox implementing the methods proposed within this paper.
(1.01 MB ZIP)
