# Supplementary material for: Identifying Prototypical Components in Behaviour Using Clustering Algorithms
Source: PLoS One. 2010 Feb 22;5(2):e9361. doi: 10.1371/journal.pone.0009361 (PMC2825265; doi:10.1371/journal.pone.0009361)
Supplement: Supporting Material S1 — Artificial data set and matlab function toolbox implementing the methods proposed within this paper. (1.01 MB ZIP) [file pone.0009361.s001.zip › pbcClustering/Kml/KmlLib/kmlocal-1.7.1.tar!/kmlocal-1.7.1/doc/kmlocal-doc.pdf]

KMlocal: A Testbed for k-means Clustering Algorithms

David M. Mount*
Department of Computer Science and
Institute for Advanced Computer Studies
University of Maryland
College Park, Maryland 20742
Email: mount@cs.umd.edu.

Version: 1.7
August 10, 2005

1 Introduction

This is a collection of programs for performing k-means clustering based on local search. In k-means clustering we are given a set of n data points in d-dimensional space and an integer k, and the problem is to determine a set of k points, called centers, so as to minimize the mean squared distance from each data point to its nearest center, called the *average distortion*.

A popular algorithm for doing k-means clustering is called the *k-means algorith*, or *Lloyd's algorithm*. Lloyd's algorithm is based on the simple observation that the optimal placement of a center is at the centroid of the associated cluster. Given any set of k centers Z, for each center $z \in Z$, let $V(z)$ denote its *neighborhood*, that is, the set of data points for which z is the nearest neighbor. Each stage of Lloyd's algorithm moves every center point z to the centroid of $V(z)$ and then updates $V(z)$ by recomputing the distance from each point to its nearest center. These steps are repeated until some convergence condition is met.

However, Lloyd's algorithm can get stuck in locally minimal solutions that are far from the optimal. For this reason it is common to consider heuristics based on *local search*, in which centers are swapped in and out of an existing solution (typically at random). Such a swap is accepted if it decreases the average distortion, and otherwise it is ignored. It is also possible to combine these two approaches (Lloyd's algorithm and local search), producing a type of hybrid solution. There are many variants on these themes.

This program provides a number of different algorithms for doing k-means clustering based on these ideas and combinations. Further information can be found in the following paper.

> T. Kanungo, D. M. Mount, N. Netanyahu, C. Piatko, R. Silverman, and A. Y. Wu, "A Local Search Approximation Algorithm for k-Means Clustering" *Proc. of the 18th ACM Symp. on Computational Geometry*, 2002, 10–18.

*Copyright (c) 2002–2005 University of Maryland and David Mount. All Rights Reserved. Partially supported by the National Science Foundation under grant CCR-0098151.

It is also available from

http://www.cs.umd.edu/~mount/pubs.html

2 Compilation

Let us assume that you are in the kmlocal root directory, from which the subdirectories src, bin, and test branch off. To start, you can compile the kmltest program by entering (from the root directory) "make". This is set up for the g++ compiler, version 2.7.2 or higher on Solaris. It will probably generate a number of error messages if you try it from another compiler or platform. The executable binary will be left in the file bin/kmltest.

3 Overview of the Algorithms

There are three different procedures for performing k-means, which have been implemented here. The main issue is how the neighbors are computed for each center.

Lloyd's: Repeatedly applies Lloyd's algorithm with randomly sampled starting points.

Swap: A local search heuristic, which works by performing swaps between existing centers and a set of candidate centers.

Hybrid: A more complex hybrid of Lloyd's and Swap, which performs some number of swaps followed by some number of iterations of Lloyd's algorithm. To avoid getting trapped in local minima, an approach similar to simulated annealing is included as well.

EZ_Hybrid: A simple hybrid algorithm, which does one swap followed by some number of iterations of Lloyd's.

All the algorithms are based around a generic local search structure. The generic algorithm begins by generating an initial solution curr and saving it in best. These objects are local to the KMlocal structure. The value of curr reflects the current solution and best reflects the best solution seen so far. The generic algorithm consists of some number of basic iterations, called stages. Each stage involves the execution of one step of either the swap heuristic or Lloyd's algorithm. Each of the algorithms differ in how they apply these stages.

Stages are grouped into runs. Intuitively, a run involves a (small) number of stages in search of a better solution. A run might end, say, because a better solution was found or a fixed number of stages have been performed without any improvement. After a run is finished, we check to see whether we want to accept the solution. Presumably this happens if the cost is lower, but it may happen even if the cost is inferior in other circumstances (e.g., as part of a simulated annealing approach). Accepting a solution means copying the current solution to the saved solution. In some cases, the acceptance may involve reseting the current solution to a random solution.

There are some concepts that are important to run/phase transitions. One is the maximum number of stages. Most algorithms provide some sort of parameter that limits the number of stages

that the algorithm can spend in a particular run (before giving up). The second is the relative distortion loss, or *RDL*. (See also KMterm.h.) The RDL is defined:

$$\text{RDL} = \frac{\text{initDistortion} - \text{currDistortion}}{\text{initDistortion}}.$$

Note that a positive value indicates that the distortion has decreased. The definition of "initDistortion" depends on the algorithm. It may be the distortion of the previous stage (RDL = consecutive RDL), or it may be the distortion at the beginning of the run (RDL = accumulated RDL).

3.1 Lloyds

This is Lloyd's algorithm with random restarts The algorithm is broken into phases, and each phase is broken into runs. Each phase starts by sampling center points at random. Each run is provided two parameters, a maximum number of runs per stage (max_run_stage) and a minimum accumulated relative distortion loss (min_accum_rdl). If the accumulated RDL for the run exceeds this value, then the run ends in success. If the number of stages is exceeded before this happens, the run ends in failure. The phase ends with the first failed run.

3.2 Swap

This algorithm iteratively changes centers by performing swaps. Each run consists of a given number (max_swaps) executions of the swap heuristic.

3.3 EZ_Hybrid

This implements a simple hybrid algorithm (compared to the full hybrid). The algorithm performs only one swap, followed by some number of iterations of Lloyd's algorithm. Lloyd's algorithm is repeated until the consecutive RDL falls below a given threshold.

A stage constitutes one invocation of the Swap or Lloyd's algorithm. A run consists of a single swap followed by a consecutive sequence of Lloyd's steps. A graphical representation of one run is presented below. The decision to make another pass through Lloyd's is based on whether the relative improvement since the last stage (consecutive relative distortion loss) is above a certain fixed threshold (min_consec_rdl).

3.4 Hybrid

This implements a more complex hybrid algorithm, which combines both of swapping and Lloyd's algorithm with a variant of simulated annealing. The algorithm's execution is broken into the following different processes: one swap, a consecutive sequence of Lloyd's steps, and an acceptance test. If we pass the acceptance test, we take the resulting solution, and otherwise we restore the old solution.

The decision to perform another Lloyd's step or go on to acceptance is based on whether the relative improvement since the last stage (consecutive relative distortion loss) is above a certain fixed threshold (min_consec_rdl). If the resulting solution is better than the saved solution, then we

accept it. Otherwise, we use the simulated annealing decision choice (described below) to decide whether to accept it. The choice to accept a poorer solution occurs with probability

$$\exp\left(\frac{\text{RDL}}{T}\right),$$

where RDL is the relative distortion loss (relative to the saved solution), and T is the current temperature. Note that if RDL > 0 (improvement) then this quantity is > 1, and so we always accept. The temperature value T is a decreasing function of the number of the number of stages. It starts at some initial value T_0 and decreases slowly over time. Rather than using the standard (slow) Boltzman annealing schedule, we use the following fast annealing schedule, every L stages we set $T = T_F \cdot T$, where:

L **(temp_run_length):** is an integer parameter set by the user. (Presumably it depends on the number of centers and the dimension of the space.)

T_F **(temp_reduc_factor):** is a real number of the form $1 - x$, for some small x.

The initial temperature T_0 is a tricky parameter to set. The user supplies a parameter p_0 (init_prob_accept), the initial probability of accepting a random swap. However, the probability of acceting a swap depends on the given RDL value. To estimate this, for the first L runs we use p_0 as the probability. Over these runs we compute the average rdl value. Once the first L runs have completed, we set T_0 so that:

$$\exp\left(-\frac{\text{avgRDL}}{T_0}\right) = p_0.$$

or equivalently

$$T_0 = -\frac{\text{avgRDL}}{\ln p_0}.$$

4 The Kmltest Driver Program

Kmltest is a driver for testing and evaluating various algorithms for the k-means problem, for point clustering in multi-dimensional spaces. It allows the user to generate or input data sets, to specify the number of centers and generate or input their initial positions, and then to run one of a number of k-means procedures. The test program is run as follows:

```
kmltest < test_input > test_output
```

where the `test_input` file contains a list of directives as described below. Directives consist of a directive name, followed by list of arguments (depending on the directive). Arguments and directives are separated by white space (blank, tab, and newline). String arguments are not quoted, and consist of a string of nonwhite characters. A character "#" denotes a comment. The following characters up to the end of line are ignored. Comments may only be inserted between directives (not within the argument list of a directive).

4.1 Basic operations

The test program can perform the following operations. How these operations are performed depends on the options which are described later.

4.1.1 Data Generation

read_data_pts ⟨file⟩

Create a set of data points whose coordinates are input from file ⟨file⟩. Prior to this, data_size must be set. At most data_size points will be read from the file. The actual number of points in the file may be less.

gen_data_pts

Create a set of data points whose coordinates are generated from the current point distribution.

4.2 Running k-means

run_kmeans ⟨method⟩

Apply k-means clustering to the current point set and clusters. The string specifies the desired version of k-means. These include:

lloyd – runs Lloyd's algorithm using the filtering algorithm.
swap – runs the swap heuristic.
hybrid – runs Lloyd's algorithm and the swap heuristic in alternating steps.
EZ-hybrid – a simpler version of hybrid. One swap followed by some number of Lloyd's.

See Section 3 for further details.

4.3 Miscellaneous

Note that in these commands, the string arguments may have no embedded blanks.

title ⟨string⟩

A title printed to the output file.

print ⟨string⟩

Outputs a string to console (cerr).

get_distortion

Computes and prints distortion (the sum of squared distances for the current centers). Note that the k-means algorithms compute and print the distortion for all stages but stage 0, if the stats level is set to "stage."

4.4 Options

How the above operations are performed depends on a set of options. If an option is not specified, a default value is used. An option retains its value until it is set again. String inputs are not enclosed in quotes, and must contain no embedded white space.

4.4.1 Options affecting nearest neighbor search

split_rule ⟨type⟩

Type of splitting rule to use in building the search tree. Choices are:

kd – optimized kd-tree
fair – fair split
midpt – midpoint split
sl_midpt – sliding midpt split
sl_fair – sliding fair split
suggest – authors' choice for best

The default is "suggest." See the file kd_split.cc for more detailed information. (Currently this is ignored!)

bucket_size ⟨int⟩

Bucket size, that is, the maximum number of points stored in each leaf node.

4.4.2 Options affecting data and query point generation

kcenters ⟨int⟩

Number of centers. Default = 5.

dim ⟨int⟩

Dimension of the space.

seed ⟨int⟩

Seed for random number generation.

data_size ⟨int⟩

Number of data points to generate for gen_data_pts points and the maximum number of data points to be read for read_data_pts. If this exceeds max_data_size, then max_data_size is incremented to match this value. Default = 100.

std_dev ⟨float⟩

Standard deviation (used in gauss, and clustered distributions). This is the "small" distribution for clus_ellipsoids. Default = 1.

std_dev_lo ⟨float⟩, std_dev_hi ⟨float⟩

Low and high standard deviations (used in clus_ellipsoids). Default = 1.

corr_coef ⟨float⟩

Correlation coefficient (used in co-gauss and co_lapace distributions). Default = 0.05.

colors ⟨**int**⟩

Number of color classes (clusters) (used in the clustered distributions). Def. = 5.

new_clust

Once generated, cluster centers are not normally regenerated. This is so that both centers and data points can be generated using the same set of clusters. This option forces new cluster centers to be generated with the next generation of either data or center points.

max_clus_dim ⟨**int**⟩

Maximum dimension of clusters (used in clus_orth_flats and clus_ellipsoids). Default = 1.

distribution ⟨**string**⟩

Type of input distribution

uniform – uniform over cube $[-1, 1]^d$.
gauss – Gaussian with mean 0
laplace – Laplacian, mean 0 and var 1
co_gauss – correlated Gaussian
co_laplace – correlated Laplacian
clus_gauss – clustered Gaussian
clus_orth_flats – clusters of orth flats
clus_ellipsoids – clusters of ellipsoids
multi_clus – multi-sized clusters

See the file rand.cc for further information.

replacement ⟨**string**⟩

Sampling option for sample_centers.

on – sample with replacement
off – sample without replacement

4.4.3 Options affection general program behavior

stats ⟨**string**⟩

Level of statistics output

silent = no output,
exec_time += execution time only
summary += summary of complete k-means
stage += summary of each stage
trace += show everything as it happens.

print_points ⟨**string**⟩

Print the points after reading or generating them. The argument is either “yes” or “no”. Default = “no”.

show_assignments ⟨string⟩

After running the clustering algorithm, print the indices of the center point to which each data point has been assigned along with its distance to this center. The argument is either "yes" or "no". Default = "no".

dump ⟨file⟩

Dump summary to ¡file¿ (for analysis by some other program).

validate ⟨string⟩

Validate experiment and compute average error. Since validation causes exact nearest neighbors to be computed by the brute force method, this can take a long time. Valid arguments are:

yes (Not implemented!) turn validation on
no turn validation off

4.4.4 Options affecting termination

The way of controlling the program's termination is to specify the maximum number of stages. (In theory, a better way would be to determine when the algorithm has converged, but this seems to be a very complex task to me.) Each time the algorithm moves the center points and recomputes the distortion constitutes a stage. The maximum number of stages is based on the number of data points n (data_size) and the number of centers k (kcenters) and four coefficients, (a, b, c, d), using the following formula:

$$\text{MaxTotalStages} = a + (b \cdot k + c \cdot n)^d$$

If the final result is 0, then the algorithm runs without terminating.

max_tot_stage ⟨4 × float⟩

Maximum total stages for given as parameters. Default: $\langle 0, 0, 0, 0 \rangle$.

4.4.5 Options used in Lloyd's Algorithm and Hybrid Algorithms

damp_factor ⟨float⟩

A dampening factor in the interval (0,1]. The value 1 is the standard Lloyd's algorithm. Otherwise, each point is only moved by this fraction of the way from its current location to the centroid. Default: 1

min_accum_rdl ⟨float⟩

This is used in Lloyd's algorithm algorithm which perform multiple swaps. When performing p swaps, we actually may perform fewer than p. We stop performing swaps, whenever the total distortion (from the start of the run) has decreased by at least this amount. Default: 0.10

max_run_stage ⟨int⟩

This is used in Lloyd's algorithm. A run terminates after this many stages. Default: 100

4.4.6 Options specific to the Swap algorithm

max_swaps ⟨int⟩

Maximum swaps at any given stage. Default: 1

4.4.7 Options specific to the hybrid algorithms

min_consec_rdl ⟨float⟩

This is used in the hybrid algorithms. If the RDL of two consecutive runs is less than this value Lloyd's algorithm is deemed to have converged, and the run ends.

4.4.8 Options specific to the (complex) hybrid algorithm

init_prob_accept ⟨float⟩

The initial probability of accepting a solution that does not improve the distortion.

temp_run_length ⟨int⟩

The number of stages before changing the temperature.

temp_reduc_factor ⟨float⟩

The factor by which temperature is reduced at the end of a temperature run.

4.5 Example

Option directives (such as "dim," "data_size," "seed") that merely set option values are indented. Operation directives ("gen_data_pts," "run_kmeans") are not indented.

```
title Experiment_1A                       # experiment title
   stats summary                          # print summary information
   print_assignments yes                  # print final cluster assignments
   dim 2                                  # dimension 2

   data_size 5000                         # 5000 data points
   colors 30                              # ...broken into 30 clusters
   std_dev 0.025                          # ...each with std deviation 0.025
   distribution clus_gauss                # clustered gaussian distribution
   seed 1                                 # random number seed
gen_data_pts                              # generate the data points

   kcenters 20                            # place k=20 center points
   distribution uniform                   # ...uniformly distributed
   seed 2
gen_centers                               # generate initial center points

   lloyd_max_tot_stage 20 0 0 0           # terminate Lloyd's after 20 stages
print Running-lloyd's
run_kmeans lloyd                          # run using Lloyd's algorithm
```

```
   seed 2                              # use same seed
gen_centers                            # regenerate same center points
   max_swaps 3                         # at most 3 swaps
   swap_max_tot_stage 0 3 0 2          # at most 3*k^2 = 1200 stages
   swap_max_run_stage 50 0 0 0         # at most 50 stages per run
   swap_min_run_gain 0.02              # stop run if distortion drops 2%
print Running-swap
run_kmeans swap                        # run using swap heuristic
```

5 Sample Program

Although KMlocal is not a library, it is possible to invoke the various algorithm directly from program. The algorithm is designed around a collection of C++ objects. The include the following:

KMdata: (KMdata.h) This object stores the data points. The constructor is given the dimension and the number of points to allocate. If P is an object of this type, then $P[i][d]$ refers to the dth coordinate of the ith point.

One the key elements to the efficiency of the algorithms presented is the use of the filtering algorithm for computing the nearest cluster center for each data point. This requires the construction of a data structure called a kc-tree. This tree is constructed by the method `P.buildKcTree()`, which should be done prior to running any of the clustering algorithms.

KMfilterCenters: (KMfilterCenters.h) This object stores the center points (in a manner that makes the use of the filtering algorithm possible). The constructor is passed two arguments, the desired number of center points k, and the associated data points.

On completion of the execution of the clustering algorithm, the centers are stored in this structure. It supports a method `print()`, which prints the centers to the standard output, and method `getDist()`, which returns the total distortion.

KMlocal: (KMlocal.h) There are currently four different clustering algorithms supported. They are all designed around a common local search template, called KMlocal. This is a generic template, and so cannot be invoked directly. The following derived objects can be invoked:

KMlocalLloyds: Repeated Lloyd's algorithm.
KMlocalSwap: The swap heuristic.
KMlocalEZ_Hybrid: A simple hybrid, which simply alternates Lloyd's algorithm and the swap algorithm.
KMlocalHybrid: A more complex hybrid algorithm, which involves simulated annealing.

These algorithms are described in Section 3, above.

The constructor to each algorithm is passed two things, the KMfilterCenter structure for the center points and the KMterm structure (described below), which specifies the termination conditions. It supports the method `execute()`, which initializes the center points to random positions, executes the clustering algorithm, and returns with the center structure modified to hold the final centers.

KMterm: (KMterm.h) Each of the local search algorithms consists of some number of incremental movements of the center points, called *stages*. Stages are grouped into longer organizational units called *runs*, and runs are grouped into longer units called *phases*. The meaning of the transition from runs to phases depends on the individual algorithm. Intuitively, a run involves a search for a better solution, through some local search operations. If this search results in a better solution, the run is said to be successful. A phase consists of a series of consecutive successful runs. When a run is unsuccessful, the phase ends.

The definition of when a run and a phase is complete depends on a number of parameters. These parameters are stored in the KMterm object. See the files KMterm.h and KMlocal.h for more detailed explanation of their exact meaning.

One important parameter in the termination condition is the maximum total stages. All the current algorithms simply execute until reaching this number of stages. It is defined by a set of four parameters (a, b, c, d). This was described earlier in Section 4.4.4. These are the first four parameters in the constructor for KMterm.

A minimal sample program is given below. It generates a set of random data points (*dataPoints*). This is done using a function *kmUniformPts*, which generates a set of uniformly distributed points in the cube $[-1, 1]^d$, where d is the dimension. It then constructs a kc-tree for these points. Next, it generates a center point structure (*ctrs*). It declares a local search algorithm (*kmAlg*) with which to perform the clustering. In this case it is the repeated Lloyd's algorithm, but the commented code indicates the possible choices for the other clustering algorithms. It creates KMterm object, which (in addition to a number of cryptic options) requests that the algorithm be run for 100 stages (given by the first four parameters being $(100, 0, 0, 0)$). It executes this algorithm, and prints the resulting distortion and center points. (This file can be found in `src/kmlminimal.cpp`. A more complete sample program can be found in `src/kmlsample.cpp`.)

```
#include <cstdlib>                      // C standard includes
#include <iostream>                     // C++ I/O
#include <string>                       // C++ strings
#include "KMlocal.h"                    // k-means algorithms

using namespace std;                    // make std:: available

// execution parameters (see KMterm.h and KMlocal.h)
KMterm  term(100, 0, 0, 0,              // run for 100 stages
             0.10, 0.10, 3,             // other typical parameter values
             0.50, 10, 0.95);

int main()
{
    int         k       = 4;                    // number of centers
    int         dim     = 2;                    // dimension
    int         nPts    = 20;                   // number of data points

    KMdata dataPts(dim, nPts);                  // allocate data storage
    kmUniformPts(dataPts.getPts(), nPts, dim);  // generate random points
    dataPts.buildKcTree();                      // build filtering structure
```

```
    KMfilterCenters ctrs(k, dataPts);           // allocate centers
                                                // run the algorithm
    KMlocalLloyds       kmAlg(ctrs, term);      // repeated Lloyd's
    // KMlocalSwap      kmAlg(ctrs, term);      // Swap heuristic
    // KMlocalEZ_Hybrid kmAlg(ctrs, term);      // EZ-Hybrid heuristic
    // KMlocalHybrid    kmAlg(ctrs, term);      // Hybrid heuristic
    ctrs = kmAlg.execute();                     // execute
                                                // print number of stages
    cout << "Number of stages: " << kmAlg.getTotalStages() << "\n";
                                                // print average distortion
    cout << "Average distortion: " << ctrs.getDist(false)/nPts << "\n";
    ctrs.print();                               // print final centers

    return EXIT_SUCCESS;
}
```

The output of the minimal sample program when run on a Sun 5 running Solaris 5.8 is shown below.

```
Number of stages: 100
Average distortion: 0.0806688
       0        [ 0.538642 -0.747656 ] dist = 0.143903
       1        [ 0.058456 -0.350953 ] dist = 0.0607307
       2        [ 0.333405 0.368641 ] dist = 0.958262
       3        [ -0.677253 -0.534964 ] dist = 0.450481
```

The data used as input to this minimal program is generated randomly, and so the final result depend on your system's random number generator. Different systems will likely produce different results. For example, the same program when compiled on a PC under Microsoft Visual Studio.NET, produced the following very different output.

```
Number of stages: 100
Average distortion: 0.137674
       0 [ 0.767327 0.301355 ] dist = 0.999582
       1 [ -0.790716 0.392132 ] dist = 0.534576
       2 [ -0.215044 -0.942462 ] dist = 0.369202
       3 [ -0.200438 0.495125 ] dist = 0.850113
```

6 How Do I Get Started Quickly?

If you have some data that you wish to cluster, I would suggest starting with the program `src/kmlsample.cpp` and modifying it for your purposes. The parameters that you will have to change are the desired number of clusters (`k`), the dimension (`dim`), the maximum number of points (`maxPts`). You should set the number of iteration stages (`stages`) to a value that you feel is large enough to guarantee good convergence. A value on the order of 100–500 should be sufficient for most instances. Larger values may be needed for higher dimensional or hard to cluster data sets. You will also need to modify the section of the program that inputs the points.

This program tries all four of the various clustering algorithm. You should see which produces the smallest distortion for your data. Based on our experience, the algorithm that has the best

overall performance is KMlocalHybrid. Finally, remove all the calls to the clustering algorithms, except for the final one you wish to use.

If you are using a PC running Microsoft Windows, precompiled versions of `kmlminimal`, `kmlsample`, and `kmltest` can be found in the directory `KMLwin32/bin`. The solution and project files can be found in `KMLwin32` as well, for compilation using Microsoft Visual Studio.NET (under Visual C++).
